# Detection of Beta-Glucan Contamination in Nanotechnology-Based Formulations

**DOI:** 10.3390/molecules25153367

**Published:** 2020-07-24

**Authors:** Barry W. Neun, Edward Cedrone, Timothy M. Potter, Rachael M. Crist, Marina A. Dobrovolskaia

**Affiliations:** Nanotechnology Characterization Lab, Cancer Research Technology Program, Frederick National Laboratory for Cancer Research Sponsored by the National Cancer Institute, Frederick, MD 21702, USA; neunb@mail.nih.gov (B.W.N.); edward.cedrone@nih.gov (E.C.); pottert@mail.nih.gov (T.M.P.); cristr@mail.nih.gov (R.M.C.)

**Keywords:** immunology, beta-glucans, contamination, drug safety, nanoparticles, sterility, endotoxin, glucatell, fungitell, factor-C-depleted Limulus amoebocyte lysate assay

## Abstract

Understanding the potential contamination of pharmaceutical products with innate immunity modulating impurities (IIMIs) is essential for establishing their safety profiles. IIMIs are a large family of molecules with diverse compositions and structures that contribute to the immune-mediated adverse effects (IMAE) of drug products. Pyrogenicity (the ability to induce fever) and activation of innate immune responses underlying both acute toxicities (e.g., anaphylactoid reactions or pseudoallergy, cytokine storm) and long-term effects (e.g., immunogenicity) are among the IMAE commonly related to IIMI contamination. Endotoxins of gram-negative bacteria are the best-studied IIMIs in that both methodologies for and pitfalls in their detection and quantification are well established. Additionally, regulatory guidance documents and research papers from laboratories worldwide are available on endotoxins. However, less information is currently known about other IIMIs. Herein, we focus on one such IIMI, namely, beta-glucans, and review literature and discuss the experience of the Nanotechnology Characterization Lab (NCL) with the detection of beta-glucans in nanotechnology-based drug products.

## 1. Introduction

Concerns about and methodologies for the detection of innate immunity modulating impurities (IIMIs) in pharmaceutical products have a long history [1]. Over the past couple of decades, significant progress has been made in detecting and quantifying product contamination with bacterial endotoxins; several in vitro and in vivo methods have been developed, extensively studied, reviewed in the literature [2,3,4,5,6,7], and addressed in guidance for industry documents by regulatory authorities worldwide [8,9,10]. The issue of potential product contamination with IIMIs received more attention when biopharmaceuticals (e.g., recombinant proteins, antibodies, and peptides) entered the generics phase [1,11]. The determination of bioequivalence for generic biotechnology products to their respective reference listed drugs (RLDs), among other tests, requires an understanding of the products’ immunogenicity, which in turn may be influenced by IIMIs. Therefore, accentuating the importance of detection of these impurities in drug products and understanding how their presence affects products’ safety profiles have become important issues for drug development [12,13].

**Figure 1:**
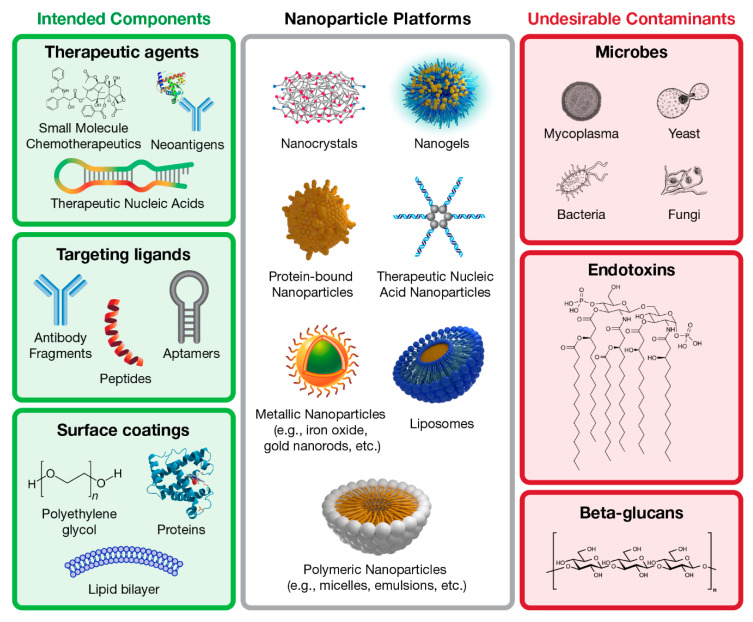
**The complex nature of nanotechnology-based therapeutics.** Nanomaterials can have a diverse chemical compositions and a broad range of physicochemical properties (e.g., size, charge, and surface functionalization), and are used for delivery of a variety of therapeutic cargoes (e.g., proteins, peptides, antibodies, and aptamers). The diverse nature of these materials, coupled with complex manufacturing procedures, makes nanomaterials prone to contamination with microbial components (e.g., endotoxin, beta-glucans, and flagellin) that act as innate immunity modulating impurities, thereby confounding the results of both efficacy and safety studies.

Nanotechnology therapeutics include a variety of chemically distinct platforms, and usually are complex in that they may contain small molecules, nucleic acids, peptides, proteins, or antibodies for targeting, as active pharmaceutical ingredients (API) or both (Figure 1). Depending on the sources of individual components and nuances of manufacturing, nanotechnology formulations may become contaminated with various IIMIs (Figure 1). However, neither the methodology for detection of IIMIs nor recognition of the potential importance of this issue for the field of nanomedicine is as well established as the current state of knowledge about endotoxins in nanoformulations. Herein, we focus on one such IIMI, namely, beta-glucans, and review the current literature on the topic, and describe the experience of the Nanotechnology Characterization Laboratory (NCL; https://ncl.cancer.gov) with the detection of beta-glucans in nanotechnology-based drug products.

## 2. Overview of Beta-Glucans

**Figure 2:**
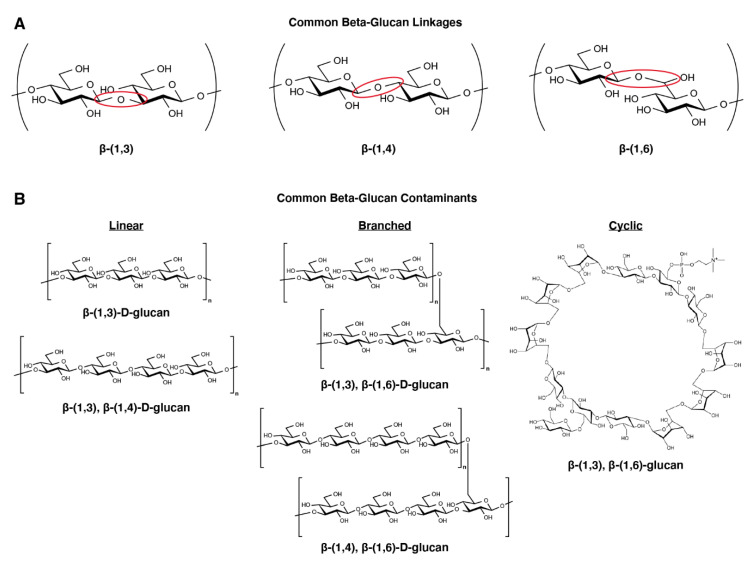
**Chemical structures of beta-glucans.** (**A**). Beta-glucans are named according to the positioning of the glycosidic linkage. The nomenclature specifies which carbons of the glucose rings are conjugated to form the polymeric structure, with carbon 1 always representing the anomeric carbon. For example, a β-(1,3) linkage conjugates the anomeric carbon of one glucose moiety to carbon 3 of another glucose moiety. β-(1,2), β-(1,3), β-(1,4), and β-(1,6) are all common linkages for beta-glucan molecules. (**B**). Beta-glucans can be linear (short and long), branched (branch-on-branch and side-chain branched) and cyclic, and can constitute polymers that utilize a single linkage positioning (e.g., β-(1,3)) or multiple linkage positionings (e.g., β-(1,3), β-(1,4)). Structures of common β-glucans within each class are given.

Beta-glucans are a family of polysaccharides with heterogeneous chemical structures (Figure 2) that are present in the cell walls of certain microorganisms (e.g., some bacteria, yeast, and fungi), algae, mushrooms and plants [14,15,16,17]. Several types of beta-glucan molecules have been described in the literature based on the positioning of the β-glycosidic bond(s)―β-(1,3); β-(1,3), β-(1,4); β-(1,3), β-(1,2), β-(1,4), β-(1,6); β-(1,2) and β-(1,3), β-(1,6)―connecting individual monomer units of d-glucose into a polymer (Figure 2A) [18]. The β-glucan polymers can take a variety of forms. Linear (short and long), branched (branch-on-branch and side-chain branched), and cyclic molecules of beta-glucans have all been identified, and can utilize a single glycosidic linkage (e.g., the linear β-(1,3)-d-glucan polymer) or multiple glycosidic linkages (e.g., the branched β-(1,3), β-(1,6)-d-glucan polymer) (Figure 2B). Alpha-glucans, which are polysaccharides of alpha-d-glucose rather than beta-d-glucose, are also commonly found in microorganisms and plants. However, α-glucans are much less pro-inflammatory as compared to β-glucans and at the moment are not considered a significant IIMI risk.

The structure (i.e., linear vs. branched vs. cyclic), conformation (i.e., triple helix vs. single helix vs. random coil), degree of branching, linkage (i.e., β-(1,3) vs. β-(1,4), etc.), and molecular weight of β-glucans all play important roles in the overall properties of the polymers, including physical aspects such as solubility, viscosity, and crystallinity, and biological aspects stemming from physiological responses to the polymers [14,16,18]. Perhaps most remarkable is the ability to activate innate immune responses depending on these physicochemical properties [14,16,18,19]. For example, a study by Noss et al. compared twelve beta-glucans with various types of β-glycosidic bonds (i.e., β-(1,3) vs. β-(1,4) vs. β-(1,6) vs. β-(1,3), β-(1,6) vs. β-(1,3), β-(1,4) vs. β-(1,4), β-(1,6)) and obtained from different sources (i.e., plant, yeast, fungi, bacteria, algae, and lichen) by using in vitro human whole blood assays to assess cytokine responses [20]. This study discovered both quantitative and qualitative differences in the cytokine profile induced by the tested beta-glucans. For example, glucans from lichens containing β-(1,4) and β-(1,6) linkages were more potent inducers of pro-inflammatory cytokines than beta-glucans with other structures or similar structures but obtained from fungi or bacteria [20]. Pro-inflammatory cytokines TNFα, IL-1β, IL-6, and IL-8 were reported as biomarkers of the beta-glucan-mediated pro-inflammatory response in human blood [20]. This observation is consistent with another study that also observed TNFα, IL-6, and IL-8 in whole blood cultures of healthy donors treated in vitro with yeast and synthetic β-(1,3)-glucans [21]. Interestingly, this study also demonstrated that linear fragments of beta-glucans were more potent at inducing Th1 responses in human blood cells in vitro than full-size molecules [21]. Another interesting observation is that synthetic beta-glucans were weaker immunostimulants than their naturally derived counterparts [21]. Even though the authors conducted experiments to rule out the potential contribution of endotoxin contamination to the cytokine response [21], it is not improbable that other undetected IIMIs present in the naturally-derived beta glucans contributed to their higher potency. Other examples demonstrating the structure activity relationships of beta glucans in different species both in vitro and in vivo are summarized in Table 1.

β-(1,3)-d-Glucans are not as immunologically potent as bacterial endotoxins but do possess immunomodulatory properties. For example, a study comparing twelve beta-glucans from various sources and with different types of glycosidic bonds reported pro-inflammatory responses to these molecules at concentrations ranging from 25 to 250 µg/mL. In contrast, the same study observed a pro-inflammatory response to bacterial lipopolysaccharides (LPS) at picogram/mL concentrations [20]. Moreover, beta-glucans were shown to exaggerate endotoxin-mediated toxicities and synergize with other immunologically-active impurities introduced into pharmaceutical products during manufacturing, thereby contributing to the adverse immune effects [32,33,34].

Pattern recognition receptors involved in the inflammatory signaling triggered by beta-glucans include Toll-like receptors (TLR2, TLR4, and TLR6) [35,36,37], dectin-1 [38,39,40], CD36 [41], scavenger receptor CD5 [42], complement receptor CR3 [43,44,45], and lactosylceramide [17]. Activation of these receptors by beta-glucan molecules results in activation of various signaling pathways, including but not limited to those triggering the activation of Src, Syk, MAP, PI3K, Akt, PKC, and IkB kinases, and key transcription factors such as NFkB, AP-1, and NFAT [46]. These receptors are differentially expressed on a wide variety of cells involved in innate immunity, such as monocytes, neutrophils, natural killer cells, granulocytes, and macrophages. Cooperation between these cells provides a coordinated response to beta-glucans.

While the exact mechanism of immunomodulation by beta-glucans is not understood, activation of the expression of TLR10 on immune cells is thought to contribute to their mechanism of action [22]. TLR10 is a member of TLR family that does not have a natural ligand but can form heterodimers with TLR1 and TLR6; it is thought to support epigenetic and metabolic reprogramming of innate immune cells in response to certain agonists present in vaccines or provided by infections and confers the enhanced response of these cells to the secondary stimulation, thereby contributing to the immunomodulation or so-called “trained” immunity [22,47].

The research on beta-glucans is of interest to pharmaceutical scientists for three reasons. First, β-(1,3)-d-glucans from dietary sources are found at low levels (<60 pg/mL) in the blood of healthy humans, whereas in patients with invasive fungal infections, these levels increase to ≥ 80 pg/mL [15,48,49,50,51]. Therefore, assessing the levels of beta-glucans in peripheral blood can serve as a diagnostic procedure for fungal infections. Second, beta-glucans are considered active ingredients in treatments aimed at improving immunity in a variety of disorders, including cancer [52]. Various forms of oral formulations are promoted worldwide as over-the-counter remedies, and some formulations are being also considered for either systemic or oral administration and undergoing clinical evaluation [53,54,55,56,57,58,59,60]. Third, β-(1,3)-d-glucans may become undesirable contaminants in pharmaceutical products, where they are inadvertently introduced during manufacturing either through certain types of personal protective equipment (PPE) suits and other cellulose-based materials (e.g., cellulose-acetate filters, cotton plugs) used in manufacturing facilities or from fungal contamination of starting materials (e.g., sucrose and sucrose-containing buffers), tools, and equipment [32,61] (Table 2). The most common sources of β-(1,3)-d-glucan contamination in pharmaceuticals are from fungi *Candida* and *Aspergillus* [48]. Moreover, the FDA immunogenicity guidance for industry suggests minimizing the levels of β-(1,3)-d-glucans in therapeutic protein formulations to decrease the immunogenicity risks of these products [62].

Since many nanotechnology platforms are not immunologically inert, understanding the presence of immunologically reactive contaminants besides endotoxin is becoming essential step in understanding the safety margins for formulations containing such materials [63]. For example, immune-mediated adverse reactions, including but not limited to infusion reactions (IRs) that occur within the first minutes to hours of systemically administered nanomedicines, are of paramount importance to patient safety. Anaphylactoid reactions, pseudoallergy, or complement-activation mediated pseudoallergy (CARPA) are common types of IRs to nanotechnology-formulated drug products. At the moment, the majority of data link nanomedicine-triggered IRs to the physicochemical properties of nanomaterials [64]. However, an IR induction by infused beta-glucans has also been reported in the literature [65,66]. Therefore, understanding the role of nanoparticle contamination with beta-glucans in IRs to nanomedicines is very important and may help to reduce both the incidence and severity of IRs. Moreover, understanding beta-glucan contamination of nanomedicines becomes particularly essential when such formulations are used for immunotherapy involving the intentional application of immune checkpoint inhibitors to enhance the immune response.

## 3. Detection of Beta-Glucans

Unlike bacterial endotoxins, the levels of β-(1,3)-d-glucan contaminants in pharmaceutical products are currently not regulated. There is no compendial standard for their detection or harmonized approach to acceptable levels. Nevertheless, there is a growing trend in the industry and among regulatory authorities worldwide to detect and quantify β-(1,3)-d-glucans, and to understand their safe levels [32,33,34].

**Figure 3:**
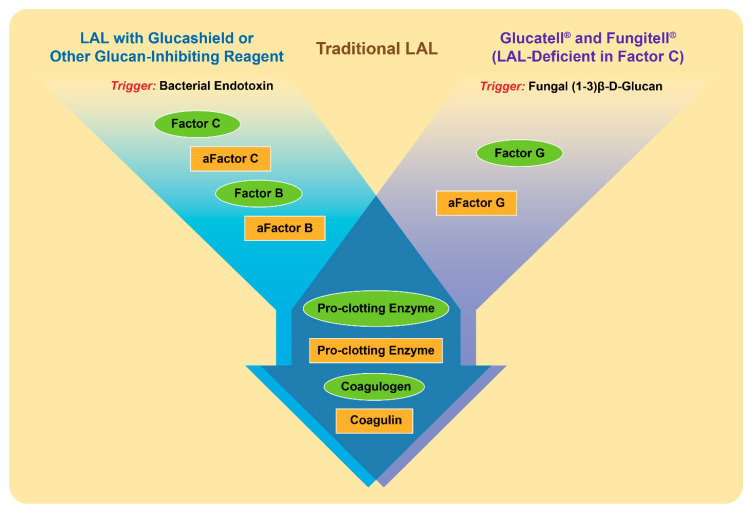
**Proteins and triggering factors of the Limulus amoebocyte lysate (LAL) Assay.** Proteins in the amoebocyte lysate of the horseshoe crab *Limulus Polyphemus* are zymogens organized in a sequential enzymatic cascade. These proteins are shown in the figure as green ovals. After the activation by either a triggering factor or preceding protein, the active form of a protein forms and is shown as a yellow rectangle; “a” at the beginning of the protein name refers to its activated state. The LAL assay can detect both endotoxins and beta-glucans. However, with slight modifications shown in the figure, the LAL may become specific to either endotoxin (left flow diagram shown in blue) or beta-glucan (right flow diagram shown in purple). These modifications are used in commercial kits (e.g., Fungitell and Glucatell) [48,72], and other commercially available reagents (e.g., Glucashield) [73].

Similarly to endotoxin, beta-glucans activate a cascade of proteins present in the lysate derived from amoebocytes of the horseshoe crab *Limulus polyphemus* and widely used in the so-called Limulus amoebocyte lysate (LAL) assay [67] (Figure 3). The raw lysate obtained from amoebocytes contains two proteins that trigger the activation of the proteolytic cascade in response to endotoxin and beta-glucans; they are factor C and factor G, respectively [67]. When the LAL assay is conducted using this lysate, the presence of factor G creates a false-positive interference of beta-glucans during endotoxin detection [68]. To overcome such interference, the assay is either modified to include glucan-blocking reagents or performed using recombinant factor C [2,69,70]. Likewise, when factor C is depleted from the lysate, the remaining factor G initiates a proteolytic cascade in response to the presence of beta-glucans [71] (Figure 3).

While the LAL assay is widely used for the detection and quantification of endotoxin contamination, its factor-C-free version, available from various manufacturers and under different tradenames, including Fungitell, Glucatell, Endosafe Nexgen-PTS, and Toxinometer MT-6500, is used to detect beta-glucans (Table 3). The Glucatell assay is used for research purposes [72], whereas the Fungitell assay is approved by the US FDA for the diagnosis of fungal infections [48]. The Fungitell assay has also been available in Europe since 2008. Toxinometer MT-6500 is another diagnostic assay that is available in the US and Asia [74]. Additional methods have also been described; they include ELISA [75] and chemical hydrolysis and enzymatic degradation-based methods [76,77,78] (Table 3). Modified LAL and ELISA assays provide higher sensitivity and are used for the detection of beta-glucans in pharmaceuticals, and in the case of diagnostic assays, in patients’ sera or plasma. The methods involving acid hydrolysis and enzymatic degradation are widely used in the food industry to quantify beta-glucans in dietary products. Additionally, methods for isolation of beta-glucans and characterization of their physicochemical properties are also available and reviewed elsewhere [79].

Studies regarding the utility of these assays for the analysis of beta-glucan contamination in engineered nanomaterials are scarce [81]. Table 4 summarizes the experience of our laboratory (https://ncl.cancer.gov) with applying the commercial Glucatell kit to screen commercial and preclinical, research-grade nanoparticle formulations for the presence of beta-glucans. The detailed experimental procedure, materials and supplies, reagent volumes, and assay incubation temperature and times are described in NCL protocol STE-4, available for download online (https://ncl.cancer.gov/sites/default/files/protocols/NCL_Method_STE-4.pdf). In our studies, the levels of beta-glucans in tested formulations varied widely from undetectable (<2.5 pg/mg) to 181,000 pg/mg of the API (Table 4). Even though some of these levels may appear high (e.g., 181,000 pg/mg), when these nanomaterials are dosed at their intended therapeutic doses, the amounts of injected beta-glucans do not exceed levels detected in the blood of healthy individuals stemming from dietary sources (see approach 4 below and reference [81] for details). Therefore, it is important to consider the data generated from beta-glucan quantification assays in the context of the dose of the nanoparticle-based product. The results obtained in our laboratory (Table 4) also demonstrate that, similarly to the experience with LAL assays [2,68,69,70,81,82], nanoparticles may interfere with beta-glucan detection and a valid response is not always observed at the lowest tested dilution. Therefore, inhibition/enhancement controls are important to verify the validity of the test-results.

A variety of approaches have been developed by researchers to overcome the test material’s interference with LAL assays for the quantification of endotoxins, should IEC reveal such interference [2,68,81,82]. One of the most straightforward approaches is to increase the dilution of the test sample. However, there are strict rules for diluting test-samples so as not to undermine the validity of the test results [9]. Specifically, in the case of endotoxin, all dilutions should not exceed the so-called maximum valid dilution (MVD) calculated according to the following formula, MVD = (EL × sample concentration)/λ, where EL is the endotoxin limit and lambda (λ) is the assay sensitivity [9]. The EL is specific to each formulation and is calculated according to the formula EL = K/M, where K is the threshold pyrogenic dose (5 EU/kg for all routes of administration except for the intrathecal route, and 0.2 EU/kg for the intrathecal route) and M is the maximum dose administered in a single hour [9]. Unlike endotoxins, the threshold pyrogenic dose of beta-glucans is not established, and it complicates the estimation of the MVD. This remains one of the current limitations in the methodology for beta-glucan detection―the lack of rules for the estimation of a valid dilution range which would allow for increased dilution to overcome nanoparticle interference with the assay. Even though the use of the lowest, non-interfering dilution is desirable, it is not always practical. Studies to understand safe levels of beta-glucans and establish their threshold dose would aid with experimental design and MVD estimation. Such studies would also help to improve current approaches for data interpretation.

## 4. Data Interpretation

No compendial procedure or criteria are currently available for the estimation of acceptable levels of β-(1-3)-d-glucans in pharmaceutical products. Below, we describe several approaches proposed by ourselves [81] and others [32,48,83,84].

*Approach 1* [83]: This is a risk-based approach that is based on the ICHQ3(R6) recommendations for establishing exposure limits to solvent impurities in drug products [85]. It includes a calculation of the permissible daily exposure (PDE) according to the following formula:

PDE = (NOAEL × weight adjustment)/(F1 × F2 × F3 × F4 × F5), where NOAEL is the no-observed-adverse-effect level derived from a toxicity study, F1 is an animal to human conversion factor, F2 is an inter-human variability factor, F3 is a subacute to chronic exposure factor, F4 is the severity of toxicity factor, and F5 is the lowest observed adverse effect level (LOAEL) to NOAEL conversion factor. The values of F1–F5 factors are 5, 10, 10, 2, and 10 for F1, F2, F3, F4, and F5, respectively.

*Approach 2* [48,84]: According to this approach, the levels of beta-glucans should remain in the range of endogenous levels. The endogenous level in a healthy individual is less than 60 pg/mL.

*Approach 3* [83]: This is a case-by-case approach that considers both PDE and endogenous levels. This approach considers individual characteristics of the product, such as format (IgG, IgE, fusion protein, etc.), origin (human or non-human), and immune-modulatory mechanism of action, and indication, the immunological status of the patient population, route, and frequency of administration.

*Approach 4* [81]: This approach estimates the dose of beta-glucans that would be injected with each dose of nanomaterial and converts it to the beta-glucan quantity per milliliter of blood. It is based on several assumptions: (a) an average adult weight is 70 kg; (b) the blood volume of such an adult is 5.6 L (or 8% of the bodyweight); and (c) the entire injected dose stays in the circulation. The estimated amount of beta-glucans per one milliliter of blood is next compared to the limit (70 pg/mL) used in the clinical diagnostic Fungitell assay where beta-glucan levels are indicative of fungal infection. For example, if the level of beta-glucan is 100 pg/mL of a nanoformulation containing 1 mg/mL of API and the API dose is 1 mg/kg, then the beta-glucan dose is 100 pg/kg; 100 pg × 70 kg = 7000 pg of beta-glucan per 5600 mL of blood. After conversion to the amount per milliliter of blood, the result is 1.25 pg/mL, which is less than 70 pg/mL. Therefore, this would be considered within normal levels of beta-glucans present in the blood from dietary sources.

*Approach 5* [32]: This approach estimates that a single dose of 500 ng of beta-glucans results in a plasma concentration of ~100 pg/mL. The same reference indicates that this level is acceptable by the UK Medicines and Healthcare Products Regulatory Agency.

## 5. Conclusions and Future Directions

Increasing interest in the quantification of beta-glucans in pharmaceutical products is justified by the growing scientific evidence of the inherent immunomodulatory properties of these molecules. While a variety of methods for detection and quantification of beta-glucans are available, there is an urgent need to validate these methods and establish compendial procedures for their application to analyze the quality of nanotechnology-based drug products and vaccines. Lessons learned from the analysis of endotoxin contamination of nanomaterials could guide future research to fulfill this need. Understanding the threshold doses of beta-glucans, estimating the MVD, establishing orthogonal methodologies for verification of test results obtained using LAL-based assays, and approaches for data interpretation are among essential focus areas for future research in this field.

## Figures and Tables

**Table 1:** **Immunological properties of glucans from various sources.** The table summarizes select examples from published studies demonstrating various types of immune responses to beta-glucans. * Whenever available, a common name is provided in parentheses. ROS = reactive oxygen species; HSP = heat shock proteins; Gm^−^ = Gram-negative; N.S. = not specified; i.m. = intra-muscular; s.c. = subcutaneous.

Type of β-glucan Molecule *	Source	On Beads (B), Particulate (P) or Soluble (S)	Immunological Response	In Vitro or In Vivo	Reference
β-(1,3)-d-glucan	N.S.	N.S.	IL-6, TNFα, IL-1RA production by human monocytes	In vitro	[22]
β-(1,6)-d-glucan (pustulan)	Yeast	B	Activation of phagocytic function, production of ROS and high levels of HSP by human neutrophils	In vitro	[23]
β-(1,3)-d-glucan (laminarin)	Yeast	B	Low levels of HSP production by human neutrophils	In vitro	[23]
β-(1,4)-d-glucan	Yeast	B	Not immunostimulatory in human neutrophils as indicated by ROS and HSP levels	In vitro	[23]
β-(1,3)-d-glucan with some β-(1,6) branching at 30:1 ratio (laminarin)	Algae	S	Lymphocyte proliferation in porcine PBMC	In vitro	[24]
β-(1,3)-d-glucan with some β-(1,6) branching at 6:1 ratio (scleroglucan)	Fungi	S	Lymphocyte proliferation; TNFα and IL-10 secretion by porcine PBMC	In vitro	[24]
β-(1,3)-d-glucan unbranched (curdlan)	Gm-Bacteria	P	Lymphocyte proliferation; ROS production by monocytes and neutrophils; TNFα and IL-10 secretion by PBMC of porcine origin	In vitro	[24]
β-(1,3)-d-glucan unbranched	Algae	P	Lymphocyte proliferation; ROS production by monocytes and neutrophils; TNFα and IL-10 secretion by PBMC of porcine origin	In vitro	[24]
β-(1,3)-d-glucan with some β-(1,6) branching at 30:1 ratio	Yeast	P	Lymphocyte proliferation; ROS production by monocytes and neutrophils; TNFα and IL-10 secretion by PBMC of porcine origin	In vitro	[24]
β-(1,3), β-(1,6) branched with 10:1 or 20:1 ratio (macrogard)	Yeast	P	Lymphocyte proliferation; ROS production by monocytes and neutrophils; TNFα and IL-10 secretion by PBMC of porcine origin	In vitro	[24]
β-(1,3), β-(1,6)-d-glucan uniformly branched (Zymozan)	Yeast	P	Lymphocyte proliferation; ROS production by monocytes and neutrophils; TNFα and IL-10 secretion by PBMC of porcine origin	In vitro	[24]
β-(1,3)-d-glucan unbranched (curdlan)	Gm- Bacteria	P	Maturation of human monocyte-derived DC; Th17 differentiation and stimulation of mixed leukocyte reaction	In vitro	[25]
β-(1,3)-d-glucan unbranched (curdlan)	Gm- Bacteria	P	Secretion of IL-1β, IL-6, IL-23, IL-10, and TNFα by human PBMC	In vitro	[26]
β-(1,3), β-(1,6)-d-glucan uniformly branched (Zymozan)	Yeast	P	Secretion of IL-1β, IL-6, IL-23, IL-10, and TNFα by human PBMC	In vitro	[26]
β-(1,3)-d-glucan	Yeast	P	Antigen-specific IgG2c and potent CD4+ T-cell activation in mice after s.c. injection	In vivo	[27]
β-(1,3), β-(1,6)-d-glucan uniformly branched (Zymozan)	Yeast	P	Complement activation; foot swelling; CTL activation; antigen-specific IgG2a antibody response and potent CD4+ Th1 response in mice after i.m. injection	In vivo	[28]
β-(1,3)-d-glucan unbranched (curdlan)	N.S.	N.S.	Elevated IL-8 levels in nasal secretions of human subjects exposed via the inhalation route	In vivo	[29]
β-(1,3)-d-glucan	Yeast	N.S.	Induction of IFNγ responses in mice after oral administration	In vivo	[30]
β-(1,6)-backbone and β-(1-3)-side branches	Mushrooms	N.S.	Elevation of L-1β, IL-2, IL-4, IL-5, IL-6, IL-7, IL-8, IL-10, IL-12, IL-13, IL-17, G-CSF, GM-CSF, IFN-γ, MCP-1, MIP-1β and TNF-α levels after oral administration in healthy human donors, and in human blood cultures	In vivo and in vitro	[31]

**Table 2:** **Common sources of beta-glucan contamination in pharmaceutical products and solutions for avoiding contamination**. Assays, such as Glucatell, could be used by individual laboratories to validate materials, procedures, and environments to minimize contamination. PPE = personal protective equipment.

Source of Contamination	Solution
Cellulose-fiber containing PPE suits	Use PPE suits made of other polymers (e.g., high- density polyethylene fibers like those in DuPont^TM^ Tyvek^®^ suits)
Cotton-containing plugs in serological pipettes and tips	Use pipettes and tips with synthetic polymer-based aerosol control barriers
Cellulose-based filters	Replace cellulose-acetate filters with other filter types; prime the filter to reduce levels of eluted glucans (the number of priming cycles may vary for different filtration units and should be determined empirically).
Sucrose and sucrose-containing buffers	Screen multiple batches and select that with minimal to no contamination; Establish a reliable supply chain
Starting materials (especially of plant origin)	Screen all starting materials and use those free of contamination; Establish a reliable supply chain
Water	Screen water and use batches free of contamination; Establish a reliable water source
Fungal contamination in the laboratory environment	Perform regular microbiological monitoring of the laboratory equipment and environment to detect and eliminate fungal contamination

**Table 3:** Assays for detection of beta-glucans in biological matrices and test-materials. LAL = Limulus amoebocyte lysate assay.

Assay Type/Name	Manufacturer	Detection Range	Diagnostic (D), R&D (R), or Food (F)	Type of Assay	Reference
Biochemical/Fungitell	Associates of Cape Cod	31.25–500 pg/mL	D	Modified LAL assay based on the measurement of optical density at 405 nm	[48]
Biochemical/Glucatell	Associates of Cape Cod	5–40 pg/mL	R	Modified LAL assay based on the measurement of optical density 405 nm (kinetic) or 540 nm (end-point)	[72]
ELISA/QuickDetect™	Biovision	0.8–50 pg/mL	D&R	Sandwich ELISA detecting absorbance at 450 nm	[75]
Biochemical/ Toxinometer MT-6500	Fuji Film	6–600 pg/mL	D	Modified turbidimetic LAL assay	[74]
Biochemical/Endosafe Nexgen-PTS	Charles River	10–1000pg/mL	R	Modified LAL, cartridge-based dedicated spectrophotometric assay	[80]
Chemical&Enzymatic/β-glucan yeast & mushroom	Megazyme	1 g/100 g	F	Acid-based hydrolysis of beta-glucans, followed by enzymatic degradation and measurement of absorbance at 510 nm	[76]
Enzymatic/yeast β-glucan	Megazyme	1 g/100 g	F	Enzymatic degradation assay measuring absorbance at 510 nm	[77]
Enzymatic/ β-glucan (mixed linkage)	Megazyme	0.5 g/100 g	F	Enzymatic degradation assay measuring absorbance at 510 nm	[78]

**Table 4:** **Levels of beta-glucans in various formulations**. Three dilutions (5, 50, and 500-fold) of the stock nanomaterial were prepared in pyrogen-free water for all formulations and tested with the commercial factor-C-depleted LAL assay (Glucatell^®^) using the procedure detailed in https://ncl.cancer.gov/sites/default/files/protocols/NCL_Method_STE-4.pdf. The results were normalized to provide beta-glucan levels in picograms per milligram of active pharmaceutical ingredient (API). The spike recovery and inhibition/enhancement control (IEC) requirements for the LAL assay were used to evaluate the performance of the Glucatell assay. The IECs were prepared by spiking a known concentration of beta-glucan standard into the test sample at each dilution. A recovery of 50–200% was considered acceptable, whereas recovery outside of this range suggested nanoparticle interference; consequently, the data from dilutions demonstrating unacceptable spike recovery were considered invalid and excluded from the analysis. The data presented are from the lowest dilution that did not interfere with the assay. BLOQ = below the assay lower limit of quantification (undetectable); SPIO = superparamagnetic iron oxide; PEG = poly(ethylene glycol).

Platform	API or * Active Component	β-Glucan Conc., pg/mg API (Spike Recovery, %)	Lowest Dilution with Acceptable Spike Recovery
Nano-albumin	Paclitaxel	5.84 (123)	5
Liposome	Amphotericin	21.3 (142)	5
PEG-liposome	Doxorubicin	154 (120)	50
SPIO	Iron	10.2 (133)	50
Nanorods	* Gold	38.5 (70)	50
Polymer-Antibody-Drug Conjugate	Cisplatin	181,000 (168)	50
Polysaccharide Nanoparticles	Paclitaxel	BLOQ (104)	500
Nanogel	Nanogel	109 (56)	50
Polymeric Nanoparticle	Iodine	21.9 (59)	50
Polymeric Nanoemulsion	Propofol	117 (111)	500
Nanocrystal	Docetaxel	129 (64)	50
Polymeric Nanoparticle	miRNA	3128 (81)	50
Polymeric Micelle	Paclitaxel	1179 (62)	500
PEG-oligo(FdUMP)	FdUMP	4.5 (93)	5
Polymeric Micelle	Neoantigen	BLOQ (64)	5
